# Draft Genome Sequences of Five *Paenibacillus* Species of Dairy Origin

**DOI:** 10.1128/MRA.00971-20

**Published:** 2020-09-10

**Authors:** Atinuke M. Olajide, Shu Chen, Gisèle LaPointe

**Affiliations:** a CRIFS, Department of Food Science, University of Guelph, Guelph, Ontario, Canada; b Agriculture and Food Laboratory, Laboratory Services Division, University of Guelph, Guelph, Ontario, Canada; University of Rochester School of Medicine and Dentistry

## Abstract

*Paenibacillus* species are important spoilage organisms in the dairy industry. The genomes of five *Paenibacillus* species which were isolated from dairy products in Canada were sequenced using the Illumina MiSeq platform. Draft genomes ranging from 5.1 to 7.1 Mb with GC contents of 49 to 53% were generated.

## ANNOUNCEMENT

*Paenibacillus* is a genus of Gram-positive psychrotolerant spore-forming bacteria associated with fluid milk spoilage by causing off flavors and curdling (1). They, together with *Bacillus* species, occur regularly in dairy farm environments, processing facilities, and pasteurized milk (2). Their spore-forming nature confers resistance to high-temperature treatments, so they survive pasteurization (3). Recently, some *Paenibacillus* species were isolated from samples obtained from a cheese processing plant in Ontario, Canada. Five isolates identified as *Paenibacillus* spp. with matrix-assisted laser desorption ionization–time of flight mass spectrometry (MALDI-TOF MS) (Bruker MBT Compass 1829023 library v9) according to the manufacturer’s manual or with sequencing of the 16S rRNA gene (4) were selected for whole-genome sequencing. Paenibacillus campinasensis 3CS1, P. macerans 3CT49, P. validus 2CS3, and P. woosongensis 12CR55 were isolated from cheese curds, and P. timonensis 12ME58 was isolated from raw milk. While 3CS1, 3CT49, and 12CS3 were obtained after a spore pasteurization treatment (80°C/12 min), 12ME58 and 12CR55 were obtained after a laboratory pasteurization treatment (63°C/30 min). All isolates but 3CT49 were obtained from aerobic incubation on brain heart infusion agar (30°C/2 days). Isolate 3CT49 grew under anaerobiosis on reinforced clostridium agar (37°C/3 days) after enrichment in reinforced *Clostridium* medium for 7 days at 37°C.

After genomic DNA from one colony per species (picked from fresh plates) was obtained using the Ultraclean microbial DNA isolation kit (Qiagen, Mississauga, ON, Canada), the concentration and quality were determined via Qubit and Nanodrop, respectively. Sequencing libraries were constructed from the DNA samples using the Nextera DNA Flex library preparation kit (Illumina Canada, Vancouver, BC) following the manufacturer’s protocol. Sequencing was performed on the MiSeq platform using a MiSeq V2 reagent kit (Illumina) and paired-end cycles according to the manufacturer’s instructions. Raw sequence reads were filtered and trimmed using the MiSeq sequencer system software v3.1 and FastQC v1.0.0. A total of 17,657,241 reads were obtained, and the average length after trimming was 250 bp.

Sequences with a quality score of ≥30 were assembled into contigs and scaffolds via *de novo* assembly following an overlap-layout-consensus method using the SPAdes v3.9.0 genome assembler (5). Genome assembly quality was evaluated using the QUAST tool (from the SPAdes pipeline) to generate summary statistics (e.g., *N*_50_, largest contig length, GC content, and number of Ns per 100 kbp) (Table 1). Gene prediction, naming, and annotation were done using the NCBI Prokaryotic Genome Annotation Pipeline (PGAP) v4.10 (6). Default parameters were used for all software unless otherwise noted. Species identities were confirmed by comparing the available 16S rRNA gene sequences in the NCBI database (https://www.ncbi.nlm.nih.gov) using the BLAST service in PATRIC v3.6.6 (https://www.patricbrc.org/) with genome sequences obtained (Table 1) due to the lack of available closed genome sequences for these species. These assemblies will help expand the study of *Paenibacillus* species, further explore their genomic diversity, and develop new tools to control spoilage of dairy products caused by these psychrotolerant spore-forming organisms.

**TABLE 1:** Characteristics of draft genome sequences and accession numbers of the five *Paenibacillus* species

Species and strain	Total no. of reads	Size of genome (bp)	No. of contigs	Length of largest contig (bp)	*N*_50_^*a*^ (bp)	GC content (%)	Coverage (×)	No. of genes	No. of RNAs^*b*^	% identity with the 16S rRNA gene (comparison length [bp]) (NCBI accession no.)	GenBank accession no.	Raw read SRA accession no.
*P. campinasensis* 3CS1	1,839,718	5,362,470	112	765,696	194,562	52.3	86	4,848	116	100.0 (1,577) (AB073187.1)	WOAA00000000	SRX7225308
*P. macerans* 3CT49	2,993,442	7,085,787	108	644,494	193,195	52.8	106	6,340	120	99.0 (1,358) (AB073196.1)	WNZZ00000000	SRX7225307
*P. timonensis* 12ME58	3,093,697	5,126,083	88	484,557	211,929	52.6	151	4,735	125	98.0 (1,357) (MK734103.1)	WNZY00000000	SRX7225306
*P. validus* 2CS3	2,573,113	5,502,307	111	573,538	193,871	52.2	117	5,341	156	99.0 (1,520) (AB073203.1)	WNZX00000000	SRX7225305
*P. woosongensis* 12CR55	3,558,563	5,683,957	78	1,379,340	475,550	49.6	156	5,122	119	100.0 (1,490) (AY847463.1)	WNZW00000000	SRX7225304

a The number of N’s per 100 kbp is 0.00 for all genomes, where N is a position that could be occupied by any of the four DNA bases.

b No. of RNAs, sum of tRNAs, rRNAs, and other RNAs.

### Data availability.

The pure cultures were deposited in the Canadian Research Institute for Food Safety (CRIFS) collection. The genome sequences reported here have been deposited at DDBJ/EMBL/GenBank under the accession numbers listed in Table 1.
